# Effect of Material Composition and Environmental Condition on Thermal Characteristics of Conductive Asphalt Concrete

**DOI:** 10.3390/ma10030218

**Published:** 2017-02-23

**Authors:** Pan Pan, Shaopeng Wu, Xiaodi Hu, Gang Liu, Bo Li

**Affiliations:** 1 School of Resource and Civil Engineering, Wuhan Institute of Technology, Wuhan 430073, China; panp@wit.edu.cn; 2 State Key Laboratory of Silicate Materials for Architectures, Wuhan University of Technology, Wuhan 430070, China; wusp@whut.edu.cn (S.W.); liug@whut.edu.cn (G.L.); libo@yzu.edu.cn (B.L.); 3 College of Civil Science and Engineering, Yangzhou University, Yangzhou 225127, China

**Keywords:** conductive asphalt concrete, thermal characteristic, material composition, environmental condition, thermal constants analyzer

## Abstract

Conductive asphalt concrete with high thermal conductivity has been proposed to improve the solar energy collection and snow melting efficiencies of asphalt solar collector (ASC). This paper aims to provide some insight into choosing the basic materials for preparation of conductive asphalt concrete, as well as determining the evolution of thermal characteristics affected by environmental factors. The thermal properties of conductive asphalt concrete were studied by the Thermal Constants Analyzer. Experimental results showed that aggregate and conductive filler have a significant effect on the thermal properties of asphalt concrete, while the effect of asphalt binder was not evident due to its low proportion. Utilization of mineral aggregate and conductive filler with higher thermal conductivity is an efficient method to prepare conductive asphalt concrete. Moreover, change in thermal properties of asphalt concrete under different temperature and moisture conditions should be taken into account to determine the actual thermal properties of asphalt concrete. There was no noticeable difference in thermal properties of asphalt concrete before and after aging. Furthermore, freezing–thawing cycles strongly affect the thermal properties of conductive asphalt concrete, due to volume expansion and bonding degradation.

## 1. Introduction

Asphalt binder is a viscoelastic solid at ambient temperature, which acts as a glue to bind aggregates and mineral fillers together [1]. Since asphalt molecules consist of weak chemical bonds that could be changed easily with temperature, mechanical behavior of asphalt pavement is also strongly dependent on temperature. Such flexible pavement would be relatively “soft” and susceptible to permanent deformation under repeated traffic loading at high temperature, while it would be relatively “hard” and susceptible to thermal cracking at low temperature [2,3]. Therefore, an understanding of temperature distribution is highly essential to determine the physical properties of asphalt mixture for predicting the pavement performance (e.g., rutting resistance analysis or thermal cracking analysis) [4]. For these reasons, various 2-D/3-D steady/transient models have been developed to study the thermal response of asphalt pavement by the finite element method (FEM) [2,5,6,7] and the finite difference method (FDM) [8,9]. The thermal properties of asphalt mixture—such as the thermal conductivity, thermal diffusivity, and specific heat—have significant effects on the distribution and variation of temperature in pavement. Gui et al. proposed that increasing thermal conductivity, diffusivity, and volumetric heat capacity of pavement materials was effective to decrease the maximum temperature of pavement [10].

Apart from the rutting and cracking problems caused by high/low temperatures, there are other realistic problems related to asphalt pavement, such as snow/ice accumulating on pavement in winter, urban heat island (UHI) effect, and others. As reported in [11], pavement impact on UHI effect is a complex problem and important interactions between influencing factors such as pavement thickness, structure, material type, and albedo must be considered. Currently, asphalt solar collector (ASC) has been proposed to keep asphalt pavement in a more appropriate temperature condition and mitigate these problems, compared to conventional asphalt pavement [12]. Such novel pavement consists of a series of serpentine or parallel pipes embedded in the pavement, below the surface layer. In hot seasons, cool water circulating through the pipes can reduce the high temperature in asphalt pavement and extract/store the warmth with energy storage technologies (EST), while in cold seasons the stored heat could warm the pavement and keep the roads free of snow and ice [13]. Therefore, ASC has attracted more and more attention due to its significant functions, such as renewable energy utilization, pavement life extension, snow melting, and UHI mitigation.

A series of experimental tests and theoretical studies have been conducted to analyze the behavior (especially energy efficiency) of ASC [14,15,16,17,18]. It is recognized that thermal characteristics are one of the most critical factors that can affect the energy efficiency of an ASC [19]. Previous literatures testified that conductive asphalt concrete can facilitate the energy transfer process occurring in the pavement of ASC [12,13,15,16]. Based on Joule heating law, conductive asphalt concrete prepared by addition of conductive materials is proposed with the initial purpose of pavement snow melting and deicing during periods of snow, sleet, or freezing rain. There are three categories for the conductive materials, including powders, fibers, and solid particles [20].

In fact, conductive materials can improve not only the electrical performance, but also the thermal properties of asphalt concrete. For the issue of ASC, conductive asphalt concrete is generally prepared by aggregate and fillers (graphite, carbon black) with high thermal conductivity. Many studies have investigated the effect of conductive materials on thermal and mechanical properties of asphalt concrete [21,22,23,24,25]. A series of studies conducted by Wu et al. indicated that graphite can improve the thermal properties of asphalt concrete and enhance the workability of ASC [7,15,26]. The efficiencies of solar collection and snow melting by using conductive asphalt concrete with graphite were increased by 11.1% and 25.9%, respectively. Additionally, mechanical properties of conductive asphalt mixture with graphite has been investigated in previous research work as well. Results indicated graphite-modified asphalt concrete shows better permanent deformation resistance, fatigue resistance, and anti-aging property, but worse moisture stability, compared to plain asphalt concrete [25,27].

As a composite material, the thermal properties of asphalt mixture are strongly dependent on the corresponding properties and proportion of each raw material, including asphalt, aggregate, and mineral fillers, as well as the amount of air voids of asphalt concrete [28]. Difference in thermal properties of these basic materials would affect the thermal properties of conductive asphalt concrete, even with the same content of graphite. Sufficient understanding of effect mechanisms of different basic materials on asphalt concrete could provide an efficient way to prepare asphalt concrete with high thermal conductivity for ASC. However, the effect of different types of asphalt, aggregate, and filler on thermal characteristics of asphalt concrete is still not reported. Moreover, realistic traffic loading and environmental loading (temperature, moisture, aging, etc.) would lead to changes in performance and structure of asphalt pavement. Such variations may affect the thermal characteristics of asphalt concrete during the service period. Absolutely accurate thermal characteristics of asphalt concrete are considerably essential for not only analyzing the distribution and variation of temperature in pavement, but also evaluating the long-term performance of an ASC. However, the effect of environmental conditions on the thermal characteristics of conductive asphalt concrete is still unknown as well.

The main objective of this paper is to provide some insight into choosing the basic materials commonly used in pavement for design and preparation of conductive asphalt concrete, as well as determining the evolution of thermal characteristics affected by environmental factors for accurately assessing the performance of ASC. The thermal properties of conductive asphalt concrete were studied by the Thermal Constants Analyzer. Three types of asphalt binders, seven types of aggregate, and two types of filler were adopted to investigate the effect of material properties and compositions on thermal characteristics of asphalt concrete. Moreover, the influences of environmental conditions, including temperature, moisture, freezing–thawing cycles. And aging on thermal characteristics of asphalt concrete were analyzed as well. The freezing–thawing test was employed to simulate the couple effect of temperature and moisture on asphalt concrete. The aging process includes short-term aging test, long-term aging test, and ultraviolet radiation aging test.

## 2. Materials and Experimental

### 2.1. Materials

Three types of asphalt binders, including AH-70, AH-90, and styrene-butadiene-styrene (SBS)-modified asphalt, were obtained from Hubei Guochuang Hi-tech Material Co., Ltd. (Wuhan, China) SBS-modified asphalt was produced by AH-90 asphalt and SBS4303 modifier. SBS4303 is a star-like polymer, containing 30 wt % styrene, and the average molecular weight is 350,000 g/mol. Table 1 illustrates the physical properties of different types of asphalt binders used in this study. 

Five types of mineral aggregates, as well as demolition waste and basic oxygen furnace (BOF) slag, were involved in this research. Among mineral aggregate, basalt, granite, diabase, and limestone were provided by Agoura Stone Processing Factory, Inner Mongolia, while dacite was provided by Longxi Stone Processing Factory, (Longxi, China). BOF slag was supplied by Metallurgical Slag Corp., Wuhan Iron and Steel. Demolition waste, obtained from Dujiangyan of Sichuan Province, was processed into recycled aggregate of different sizes. Basic characteristics of aggregates were measured according to ASTM standards and EN standards. The results are listed in Table 2. 

Limestone powder—with a particle size of less than 0.075 mm, a density of 2.699 g/cm^3^, and a thermal conductivity of 2.92 W/(m·K)—was used as the mineral filler. Graphite was used as thermal conductive filler. It has a density of 2.1 g/cm^3^ and a thermal conductivity of 59.32 W/(m·K) and consists of carbon (98.9%), ash (0.2%), and iron (0.03%) by weight. Its particle size was less than 0.075 mm. 

### 2.2. Preparation of Specimens

**Figure 1:**
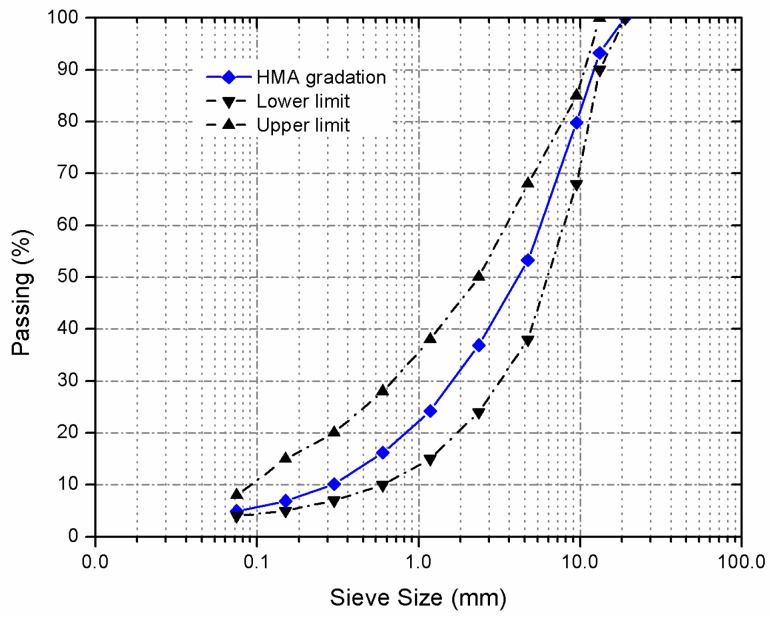
Chart of aggregate gradation.

Superpave procedure was adopted to design asphalt mixture with the maximum nominal size of 12.5 mm in this research. Volume control method was used in order to consistently maintain the volume composition of different mixtures. The target gradation of different asphalt mixtures prepared by various aggregates is shown in Figure 1. To ensure the accuracy of the results, each type of aggregate was divided into nine categories according to the sieve sizes (0.075–19 mm). The gradation curves for different mixtures could overlap the target gradation in the coordinate system of mass passing percent, and limestone powder filler was 4% by volume. Considering that only the asphalt binder type or aggregate type in each mixture was different, the names of corresponding aggregate or asphalt binder were adopted to describe asphalt mixtures.

Control and conductive asphalt mortar/mixture samples were also prepared to investigate the effect of environmental conditions on their thermal characteristics. On the one hand, the effect of temperature on thermal properties of different mortars was studied. Control asphalt mortar, which is named CAB, was prepared by the same weight as AH-70 asphalt and limestone filler. For graphite-modified asphalt mortar, which is named GMAB-40, graphite substitutes for 40% limestone filler by volume. The detailed preparation procedure was described in a previous research work [25].

Additionally, the influence of freezing–thawing cycles and aging on asphalt mixtures was analyzed. SBS-modified asphalt binder was used to prepare the control and conductive asphalt mixtures. Basalt asphalt mixtures with and without graphite were named GMAC-40 and PAC, respectively. For conductive asphalt concrete, 40% limestone filler was replaced by graphite with the same volume, which was considered an appropriate proportion to meet the requirements for mechanical properties. Design and preparation of conductive asphalt concrete were described in the study by Wu et al. [29].

### 2.3. Thermal Characteristics Analysis

The present work mainly focused on the thermal characteristics of asphalt mixture affected by material composition and environmental conditions. The diameter and height of ASC specimens are 100 mm and 60 mm, respectively. The specimen was cut into four cylindrical pieces, whose heights were 15 mm and marked by height from bottom to top. The thermal characteristics of conductive asphalt concrete were measured by the Thermal Constants Analyzer (TPS 2500S, Hot Disk, Göteborg, Sweden), which is based on the transient plane source (TPS) method following the standard ISO 22007-2:2008(E) [30]. TPS, which utilizes a plane sensor and a special mathematical model describing the heat conductivity and combines with electronics, enables the method to measure the thermal transport properties. The detailed measurement procedure was reported in previous research work [31]. In this study, six test repetitions were tested and the average value was used. The thermal conductivity and thermal diffusivity were measured by the Thermal Constant Analyzer and the specific heat was calculated as below in Equation (1).

(1) $$c_{v}=\frac{\lambda }{\alpha }$$

where *c_v_* is the specific heat, MJ/(m^3^·K); *λ* is the thermal conductivity, W/(m·K); and *α* is the thermal diffusivity, mm^2^/s.

### 2.4. Standard Aging Procedure

In this study, short-term aging (STA), long-term aging (LTA), and ultraviolet (UV) radiation aging tests were employed to investigate the effect of aging on the thermal properties of conductive asphalt mixture. The STA test and LTA test were conducted according to AASHTO PP2-1994. For the standard STA test, the asphalt mixture was spread to a height of 50 mm on a metal pan and placed in a force draft oven at 135 °C for 4 h. The standard STA test represents aging occurring until the materials are placed in the pavement. For the LTA test, the compacted specimen was prepared by placing a standard STA aged mixture in the oven at 85 °C for 5 days. The LTA test represents 5 years of in service aging [32]. For the UV aging test, Marshall specimens prepared by STA aged mixture were placed on the roof and exposed to the sunlight. A glass plane with a thickness of 3 mm was placed on the surface of specimen. The UV aging test simulates ultraviolet radiation aging that occurs during the service life. The thermal characteristics of specimen after UV aging were studied every 2 months during half a year. All the STA, LTA, and UV aged specimens were employed in a thermal properties test.

### 2.5. Freezing–Thawing Procedure

Considering the AASHTO T283 testing procedure, a modified freezing–thawing test was used in this work. After vacuum saturation for 15 min, the conditioned specimen was firstly placed in a plastic bag with 30 mL injected water. Then, the sealed plastic bag with the specimen was put in freezer with a temperature of −18 °C for 16 h. Finally, the specimen was taken out and put into a water bath to be thawed at 60 °C for 8 h. Although the freezing–thawing test method was extremely laborious and time consuming, the specimens, after different freezing–thawing cycles (1, 3, 5, 7, 10, 15, 20, 25, 30, respectively), were employed in volumetric and thermal properties tests.

## 3. Results and Discussion

### 3.1. Effect of Material Composition on Thermal Characteristics of Asphalt Concrete

#### 3.1.1. Asphalt Binder

Three types of typical asphalt binders, including AH-70, AH-90, and SBS-modified asphalt, were employed to study the effect of different asphalt binders on thermal properties of asphalt mixture. Table 3 illustrates the thermal properties of different types of asphalt binders. Asphalt binder is made up of complex hydrocarbons, such as aliphatic, aromatic, and cyclic molecules. It is known that the physical properties of asphalt binder are dependent on the proportion of chemical compositions, which could affect the thermal properties as well. As shown in Table 3, there were certain differences in thermal conductivity, thermal diffusivity, and specific heat between AH-70, AH-90, and SBS-modified asphalt binders. Thermal conductivity and specific heat of SBS-modified asphalt binder were increased by almost 10% compared to that of virgin asphalt binders. The addition of SBS modifier with higher molecular weight improved the thermal conductivity and specific heat of virgin asphalt binder of AH-90.

In order to evaluate the effect of different asphalt binders on thermal properties of asphalt mixtures, basalt was adopted to prepare asphalt mixtures with AH-70 asphalt binder, AH-90 asphalt binder, and SBS-modified asphalt binder, respectively. The thermal conductivity, thermal diffusivity, and specific heat of different asphalt mixtures are shown in Table 4. Thermal characteristics of asphalt mixtures varied with the types of asphalt. Among three types of asphalt mixtures, SBS-modified asphalt mixture acquired the highest thermal conductivity and specific heat, which was in accordance with the results in Table 3. It implied that thermal characteristics of asphalt can affect the corresponding thermal properties of asphalt mixture, even with the same aggregate gradation and the same asphalt content.

However, the differences in thermal properties of asphalt mixtures were not as significant as the differences in thermal properties of asphalts. As seen in Table 3, there was a noticeable difference in thermal properties of asphalt materials. The differences between the maximum and minimum values of thermal conductivity, thermal diffusivity, and specific heat were 13.2%, 4.2%, and 12.4% for AH-70, AH-90, and SBS-modified asphalt binders, respectively. On the other hand, as shown in Table 4, the corresponding differences in thermal conductivity, thermal diffusivity, and specific heat were 4.7%, 0.6%, and 4.1% among different types of asphalt mixtures. It is known that the weight (or volume) proportion of asphalt is relatively low in asphalt mixture. Therefore, different types of asphalt binders could not result in a significant difference in thermal properties of asphalt mixtures. 

#### 3.1.2. Aggregate

The thermal properties of asphalt mixture prepared by seven types of aggregates were measured, and the result is shown in Table 5. SBS-modified asphalt was adopted to prepare each kind of asphalt mixture. As seen in Table 5, the differences between the maximum and minimum values of thermal conductivity, thermal diffusivity, and specific heat were 36.3%, 43.4%, and 28.3%, respectively. Compared to asphalt binder, aggregate can significantly change the thermal properties of asphalt mixtures, even with the same aggregate gradation, the same kind of asphalt binder, and the same asphalt content. Generally, aggregate accounts for more than 90% by weight of asphalt mixture. The thermal properties of asphalt mixture are strongly dependent on the corresponding properties of aggregate. Therefore, different types of aggregate could result in a noticeable difference in thermal properties of asphalt mixtures.

The thermal properties of aggregate are determined by its chemical composition, structure, and surface texture. Dacite has considerably high thermal conductivity due to its high crystallization and dense structure. Consequently, asphalt concrete prepared by dacite acquired the highest thermal conductivity among the seven types of asphalt concrete in this study. Demolition waste, which has a water absorptivity of 6.9%, consists of recycled cement concrete and brick. A porous structure results in low thermal conductivity of demolition waste. Correspondingly, demolition waste mixture shows the lowest thermal conductivity. Therefore, aggregate with high thermal conductivity (e.g., dacite) is recommended to prepare the conductive asphalt mixture in order to improve the energy efficiency of an ASC.

**Figure 2:**
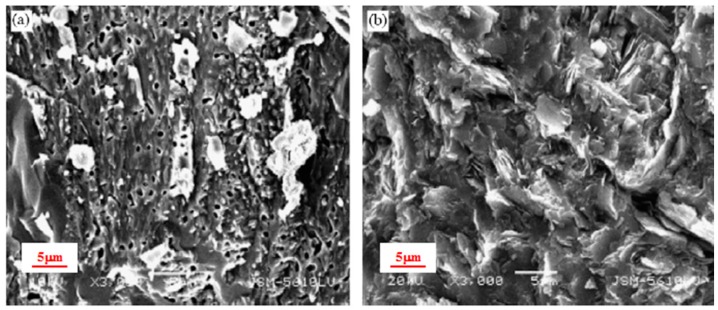
SEM images of BOF slag (**a**) and basalt (**b**).

In addition, BOF slag contains a large amount of metallic oxide. Since metal has excellent thermal conductivity, it is theoretical that BOF slag mixture should have excellent thermal conductivity as well. However, the thermal conductivity of BOF slag mixture is 1.612 W/(m·K), which is lower than that of basalt mixture (1.622 W/(m·K)), granite mixture (1.781 W/(m·K)), diabase mixture (1.717 W/(m·K)), and dacite mixture (2.123 W/(m·K)). Figure 2 illustrates that BOF slag was a kind of porous material compared to basalt. It was proved that more asphalt is required to utilize the BOF slag as aggregate for asphalt mixture [33]. Therefore, BOF slag could not improve the thermal conductivity of asphalt mixture compared to common mineral aggregates. It is concluded that the surface texture can significantly affect the thermal properties of asphalt mixtures as well. 

#### 3.1.3. Filler 

**Figure 3:**
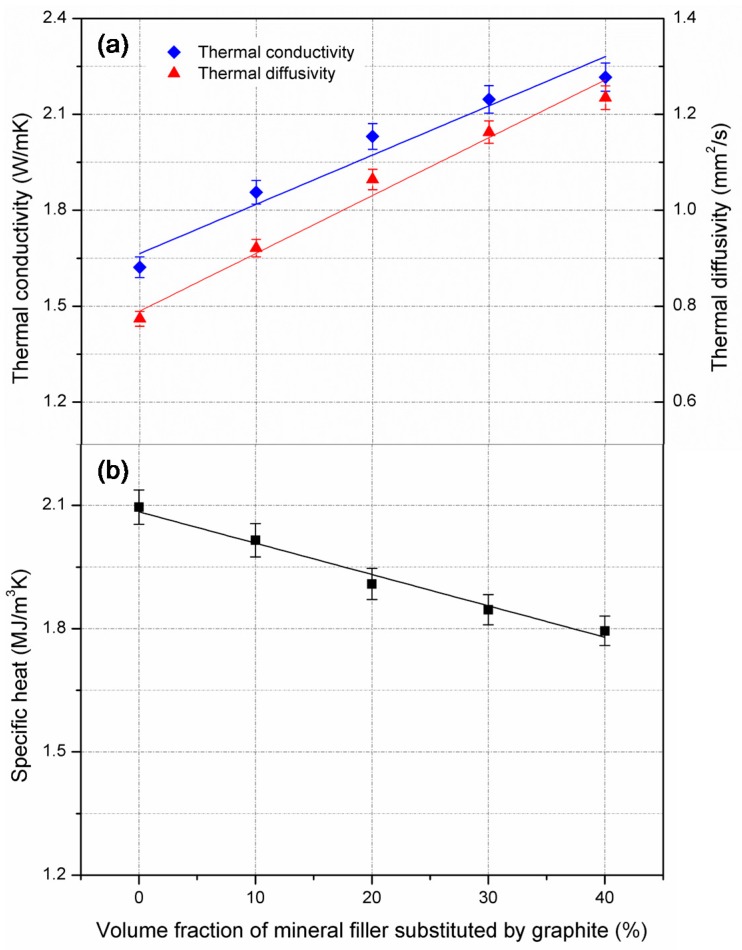
Effect of graphite on thermal characteristics of asphalt mixtures. (**a**) Thermal conductivity and diffusivity; (**b**) Specific heat.

In this study, limestone filler and graphite were employed to study the effect of filler on thermal properties of basalt asphalt mixture. Thermal conductivity, thermal diffusivity, and specific heat of specimens with different content of graphite (10%, 20%, 30%, and 40% by volume of limestone filler) were measured and the results are shown in Figure 3. As seen in Figure 3a, the thermal conductivity and thermal diffusivity of asphalt mixture tended to increase as the volume content of limestone filler replaced by graphite increased. When the content of graphite increased from 0% to 40%, the thermal conductivity of asphalt mixture increased from 1.621 W/(m·K) to 2.217 W/(m·K), and the thermal diffusivity increased from 0.774 mm^2^/s to 1.235 mm^2^/s. 

As mentioned in Section 2.2, limestone filler accounted for 4% by weight of asphalt mixture. The density of limestone is comparative to that of graphite. When graphite substituted for 40% limestone filler by volume, the content of graphite in asphalt mixture was almost 2% by weight of asphalt mixture. Such a small proportion of graphite improved the thermal conductivity by 37%, and improved the thermal conductivity of basalt asphalt mixture by 59%. In this circumstance, thermal conductivity and thermal diffusivity of basalt asphalt mixture is comparative to that of dacite mixture. As shown in Figure 3b, specific heat of asphalt mixture presented a descending trend with the increment of graphite. When graphite substituted for 10% limestone filler by volume, the specific heat of asphalt mixture was decreased by 4%, almost 0.077 MJ/(m^3^·K). The reduction of specific heat also implied that the increment of thermal diffusivity was higher than the increment of thermal conductivity, which could be explained by the mathematic relationship between the thermal characteristics described by Equation (1). 

The thermal conductivity, thermal diffusivity, and specific heat of graphite were 59.32 W/(m·K), 41.72 mm^2^/s, and 1.422 MJ/(m^3^·K), while the corresponding parameters of mineral filler were 2.92 W/(m·K), 1.49 mm^2^/s, and 1.951 MJ/(m^3^·K). The thermal conductivity and thermal diffusivity of graphite is an order of magnitude higher than that of limestone filler, while their specific heats are comparative. Therefore, graphite significantly affects the thermal conductivity and thermal diffusivity of asphalt mixture, compared to specific heat. Threshold phenomenon is generally used to explain the effect of conductive materials on electrical conductivity of asphalt mixture. A sudden change in electrical conductivity of asphalt concrete would take place with the addition of graphite [27]. Different from electrically conductive asphalt mixture, the threshold phenomenon is not suitable for graphite-modified asphalt mixture with aspect to thermal conductivity. This is because that the difference in thermal conductivity is not as significant as that in electrical conductivity. Graphite particles, which do not form the connected conductive path, also contribute to improving the thermal conductivity of asphalt mixture. Therefore, the thermal conductivity and thermal diffusivity of asphalt mixture showed a linear increase and the specific heat showed a descending trend with the increment of graphite. It could be concluded that the effect of filler on thermal characteristics of asphalt mixture is strongly dependent on not only the thermal properties of fillers (mineral filler or conductive filler), but also the amount of conductive filler.

### 3.2. Effect of Environmental Condition on Thermal Characteristics of Conductive Asphalt Concrete

#### 3.2.1. Temperature

Conductive asphalt mixture consists of asphalt binder, mineral aggregate, filler, and graphite. To better understand the effect of temperature on thermal characteristics of asphalt mixtures, it is necessary to analyze how the temperature affects the thermal properties of each material.

##### Asphalt Binder

**Figure 4:**
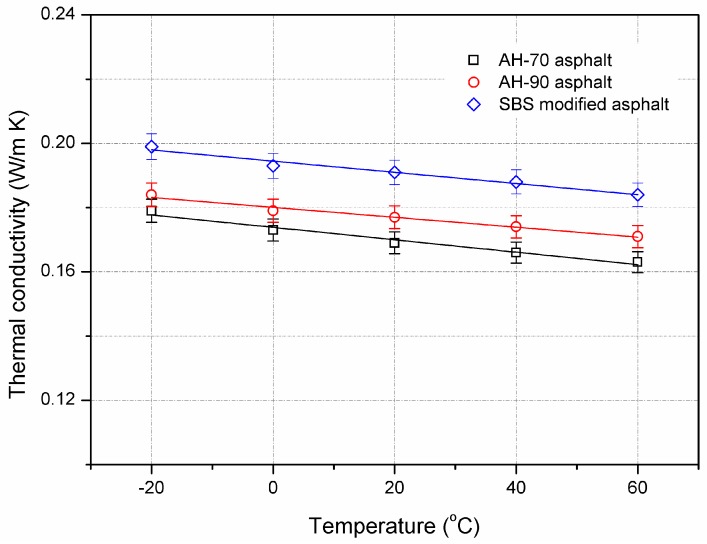
Effect of temperature on thermal conductivity of asphalt binders.

The evolution of thermal conductivity of AH-70 asphalt binder, AH-90 asphalt binder, and SBS-modified asphalt binder at different temperatures is shown in Figure 4. The test temperature ranged from −20 to 60 °C, in consideration of the service condition of asphalt pavement. The thermal conductivity of asphalts shows a linear decreasing trend with the increment of temperature. When the test temperature increased from −20 to 60 °C, the decrements of thermal conductivity of AH-70 asphalt, AH-90 asphalt, and SBS-modified asphalt were 8.9%, 7.1%, and 7.5%, respectively. This indicated that the temperature has a relatively small effect on the thermal conductivity of asphalt binder. Therefore, asphalt binder contributes little to the variation of thermal conductivity of asphalt mixture when temperature changes.

**Figure 5:**
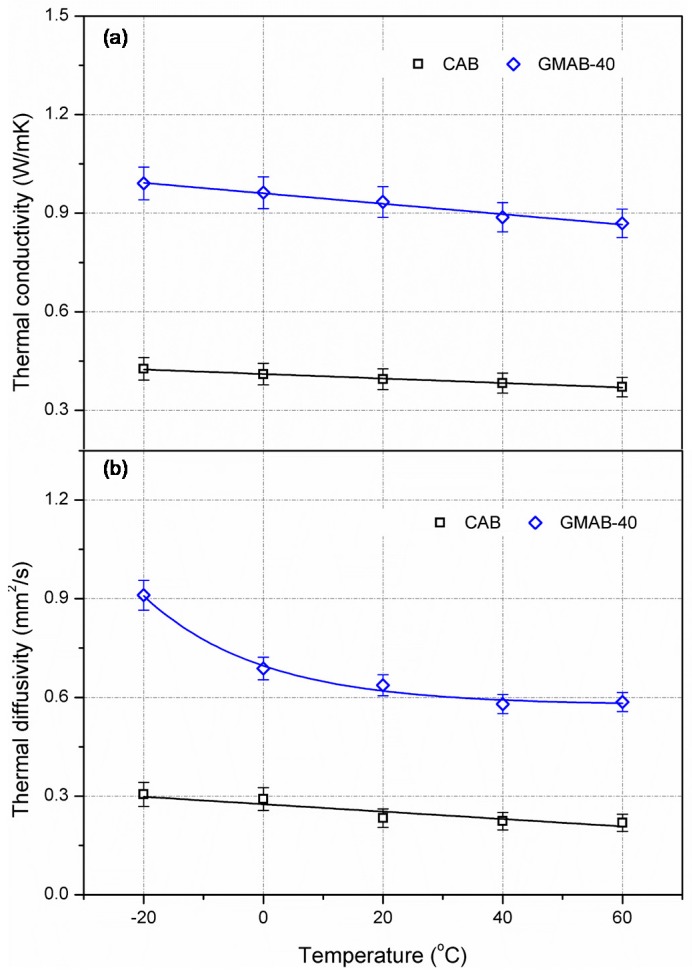
Effect of temperature on thermal characteristics of asphalt mortars. (**a**) Thermal conductivity; (**b**) Thermal diffusivity.

The effect of temperature on thermal conductivity and thermal diffusivity of asphalt mortars were analyzed and the results are shown in Figure 5. The thermal conductivity of CAB and GMAB-40 specimens tended to decrease as the test temperature increased. When the temperature increased from −20 to 60 °C, the thermal conductivity of the CAB specimen was decreased by 12.9%, from 0.426 to 0.371 W/(m·K), while the corresponding result of the GMAB-40 specimen was decreased by 12.3%, from 0.991 to 0.869 W/m·K. This implied that temperature has a comparative effect on thermal conductivity of control asphalt mortar and conductive asphalt mortar.

In addition, the thermal diffusivity of CAB and GMAB-40 mortars decreased as the temperature increased. It was noticeable that the thermal diffusivity of GMAB-40 mortar decreased rapidly in the low-temperature region. When the temperature increased from −20 to 0 °C the thermal diffusivity of the GMAB specimen was decreased by 35.7%, from 0.911 to 0.586 mm^2^/s, while the corresponding result of the CAB specimen was only decreased by 4.6%. The reason might be that the specific heat of graphite increased in a large amplitude, leading to such a rapid reduction of thermal diffusivity of GMAB-40 asphalt mortar. On the other hand, it is known that graphite shows better compatibility with asphalt and higher oil-absorption compared to mineral filler [25]. Graphite powder could transfer more free asphalt to structural asphalt. Compared to CAB asphalt mortar, the temperature effect was more significant for the GMAB-40 sample in the low-temperature region, between −20 and 0 °C. Further research is needed to be conducted on this special behavior. When the temperature ranged from 0 to 60 °C, the reduction of thermal diffusivity of the CAB and GMAB-40 specimens were 24.7% and 14.8%. This implied the thermal diffusivity of the CAB specimen was more sensitive to temperature than the conductive asphalt mortar with graphite.

##### Aggregate and Filler

The thermal conductivity process refers to the heat energy transferring inside of a substance in the form of diffusion. According to the medium, the heat-transfer mechanism falls into two categories: (i) electronic model, which depends on migration of free electrons; and (ii) phonon model, which depends on vibration wave of lattice. For nonmetallic crystal material, only the phonon model exists. The thermal conductivity can be described by Equation (2) [34].

(2) $$k=\frac{1}{3}C_{ph}\bar{c_{ph}}\lambda_{ph}$$

where *k* is the thermal conductivity, W/(m·K); *C_ph_* is the volumetric specific heat of phonon, kJ/(m^3^·K); $\bar{c_{ph}}$ is the mean speed of phonon, m/s; and *λ_ph_* is the mean free path of phonon, m. 

Asphalt is a typical kind of amorphous material, which is a mixture of hydrocarbon and non-hydrocarbon composition. Apart from asphalt, mineral aggregate, filler, and graphite are nonmetallic crystal materials and their thermal conductivity can be explained by Equation (2). According to Equation (2), thermal conductivity is proportional to specific heat and is inversely proportional to mean free path. When temperature increases, specific heat of material tends to increase, while the mean free path tends to decrease. Therefore, variation of thermal conductivity is the result of combination effect of specific heat and mean free path. 

Thermal characteristics of conductive asphalt mixture is strongly dependent on the thermal properties and proportion of each composition (e.g., asphalt, aggregate, filler, and graphite). As shown in Figure 5, temperature hardly affects the thermal properties of asphalt. Since mineral aggregate accounts for a large proportion in asphalt mixture, and graphite is an important agent to improve the thermal properties of asphalt mixture, the thermal conductivity and thermal diffusivity of asphalt mixture would follow a trend similar to aggregate and graphite. Previous studies conducted by Maqsood et al. and Cai et al. indicated that thermal conductivity of granite and graphite would show a noticeable reduction of thermal conductivity as temperature increases [35,36]. Therefore, the effect of temperature on thermal properties of asphalt mixture should be taken into consideration for accurately determining the temperature distribution for pavement, especially for performance evaluation of an ASC. Limited to laboratory conditions, thermal properties of mineral aggregate, filler, and graphite at different temperatures were not measured in the current work. Further study is also needed to examine how temperature affects the thermal properties of aggregates, limestone filler, graphite, as well as asphalt mixtures used in this study.

#### 3.2.2. Water

**Figure 6:**
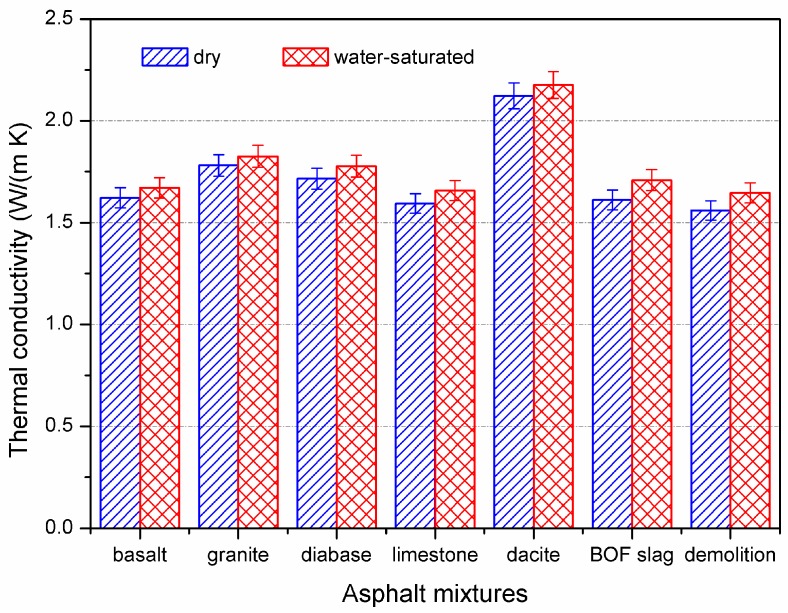
Thermal conductivity of dry and water-saturated asphalt mixtures.

During the snow-melting process of ASC, water would enter into the air void of asphalt pavement. Thermal conductivity of air is considered as 0.025 W/(m·K), while that of water is 0.602 W/(m·K). There is a significant difference in thermal conductivity between the air and the water. Therefore, it is necessary to study whether the condition of water inside affects the thermal properties of asphalt mixture. The specimens were prepared by SBS-modified asphalt binder, limestone filler, and different types of aggregate in this study. The thermal conductivities of dry specimen and water-saturated specimen were compared, and the results are shown in Figure 6. The water-saturated specimens were acquired according to standard of AASHTO T283.

Since the thermal conductivity of water is higher than that of air, the water-saturated specimen showed better thermal conductive performance than the dry specimens. For seven types of asphalt mixtures in this study, the changes of thermal conductivity ranged from 2.5% to 6.0%, which was determined by the aggregate. However, the effect of water on thermal properties of asphalt mixture was not as significant as that of temperature. This is because the air void of dense asphalt mixture is only 4%. Although the differences in thermal characteristics between air and water are noticeable, the effect of water on thermal properties of asphalt mixtures was still limited.

#### 3.2.3. Freezing–Thawing Cycles

Water would enter inside the asphalt concrete in the environment laden with moisture for a long term. Ice can be formed in negative temperature and be melted as the temperature rises above 0 °C. Multiple freezing–thawing cycles would result in volume expansion and crack occurrence of asphalt concrete. Therefore, such variation might lead to changes in thermal properties of asphalt concrete as well. In this study, the effects of freezing–thawing cycles on volumetric and thermal properties of conductive asphalt mixture and control specimens were studied. Variation of air void value was adopted to evaluate the volumetric properties of asphalt concrete before and after freezing–thawing cycles. After certain freezing–thawing cycles, the specimens were placed in a drying oven with the temperature of 60 °C until they maintained a constant weight. Thereafter, the air void value was calculated as below, in Equation (3). In this study, six test repetitions were tested and the mean value was used.

(3) $$Air\;void\;value=\left(1-\frac{\gamma_{f\_i}}{\gamma_{t}}\right)\times 100\%$$

where *γ_f_i_* is the bulk density of specimen after *i* times of freezing–thawing cycles, and *γ_t_* is the theoretical maximum density of asphalt mixture.

**Figure 7:**
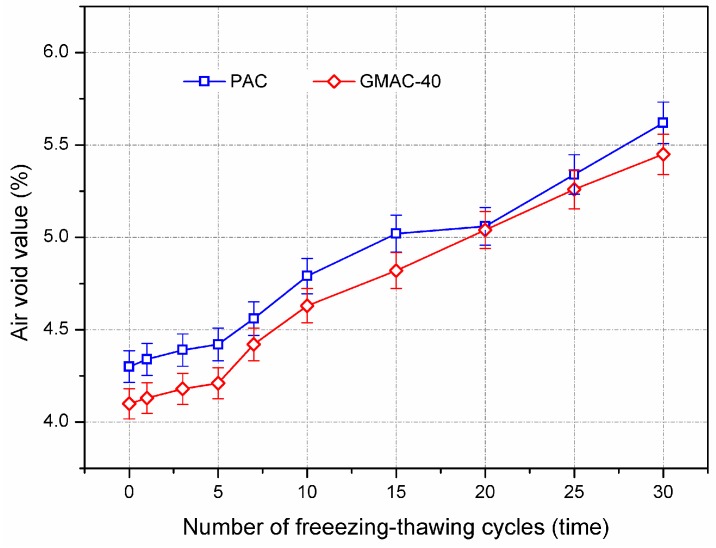
Variation of air void of asphalt mixtures after freezing–thawing cycles.

Figure 7 illustrates the evolution of the air void value of conductive and control specimens after freezing–thawing cycles. The PAC group represents the plain basalt asphalt mixture without graphite, while the GMAC-40 group represents the asphalt mixture with graphite content of 40% by volume of limestone filler. As shown in Figure 7, the air void of both control and conductive specimens tended to increase after freezing–thawing cycles. This is because the repeated freezing–thawing effect consequently caused the volume expansion of asphalt mixture, which lead to the air void increasing. Generally, higher change of air void indicated that the asphalt mixture had worse freezing–thawing resistance. There was no significant difference in air void between the control and conductive specimens at the same freezing–thawing cycles. Therefore, it could be concluded that graphite would not degrade the freezing–thawing resistance of asphalt concrete.

**Figure 8:**
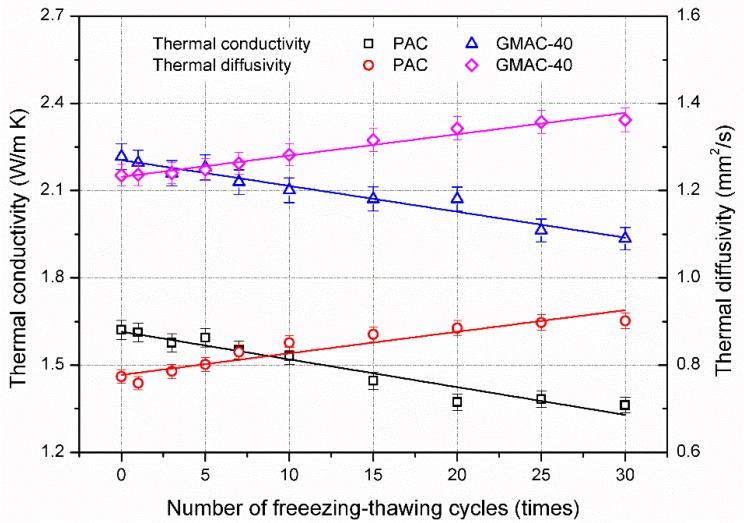
Thermal conductivity and diffusivity of asphalt mixtures after freezing–thawing cycles.

**Figure 9:**
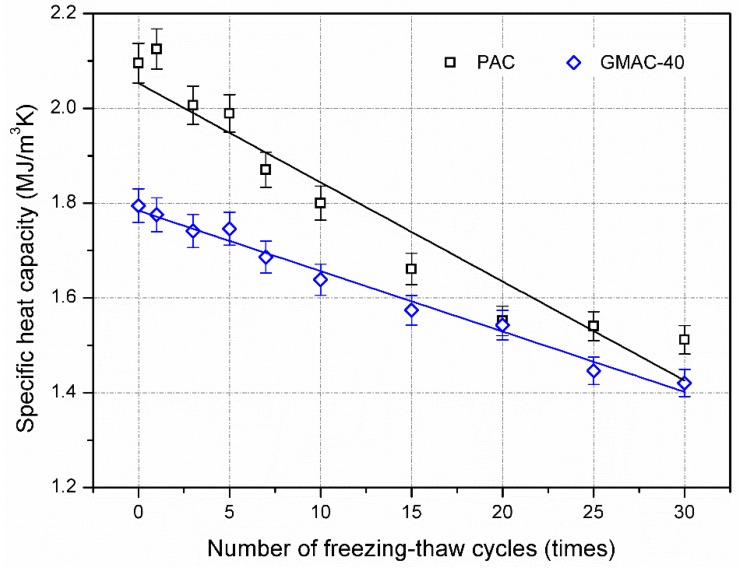
Specific heat capacity of asphalt mixtures after freezing–thawing cycles.

Figure 8 and Figure 9 illustrate the effect of freezing–thawing cycles on the thermal properties of conductive asphalt concrete and control specimens. As shown in Figure 8, the thermal conductivity of asphalt concrete tended to decrease with the addition of freezing–thawing cycles, and the thermal diffusivity increased correspondingly. After 30 freezing–thawing cycles, the thermal conductivity of control and conductive specimens decreased by 15.9% and 12.7%, respectively. In accordance with Figure 7, volume expansion caused by the freezing–thawing effect would increase the volume proportion of air void in asphalt concrete. The thermal conductivity of air is 0.025 W/(m·K), which is lower than that of asphalt (0.17 W/(m·K)) and mineral materials (2.68 W/(m·K)). Additionally, the bonding characteristic become worse during the freezing–thawing effect, leading to higher thermal contact resistance between the asphalt mortar and aggregate. Therefore, the combination of these factors reduced the thermal conductivity of asphalt concrete.

However, the thermal diffusivity of control specimen increased by 16.5%, from 0.774 to 0.902 mm^2^/s, while the thermal diffusivity of conductive specimen increased by 10.3%, from 1.235 to 1.362 mm^2^/s. The thermal diffusivity implied the ability to respond to change of environment temperature, which is a parameter used to evaluate the relationship of thermal conductivity and specific heat of material. The experimental results implied that freezing–thawing effect degraded the thermal conductivity of asphalt concrete, but simultaneously improved the thermal response ability.

Figure 9 illustrates the change in specific heat of asphalt concrete during the freezing–thawing test. The specific heat tended to decline with the addition of freezing–thawing cycles. The specific heat is strongly dependent on the energy storage capacity of material. Specific heat values for air, asphalt, and mineral material are 1 kJ/(m^3^·K), 1.7 MJ/(m^3^·K), and 2.1 MJ/(m^3^·K), respectively. The increment of air void means the volume proportion of asphalt mortar and aggregate decreased, leading to the specific heat of asphalt concrete decreasing. After 30 freezing–thawing cycles, the specific heat values of control and conductive specimens were decreased by 27.9% and 20.8%, respectively. The effect of freezing–thawing cycles on specific heat was the most significant compared to thermal conductivity and thermal diffusivity. The results in Figure 8 and Figure 9 also imply that the decreased ratio of specific heat was higher than that of thermal conductivity for conductive asphalt concrete and plain asphalt concrete, respectively. According to the relationship shown in Equation (1), the thermal diffusivity of asphalt concrete would present an increasing tendency, which is in accordance with the result in Figure 8. 

#### 3.2.4. Aging

**Figure 10:**
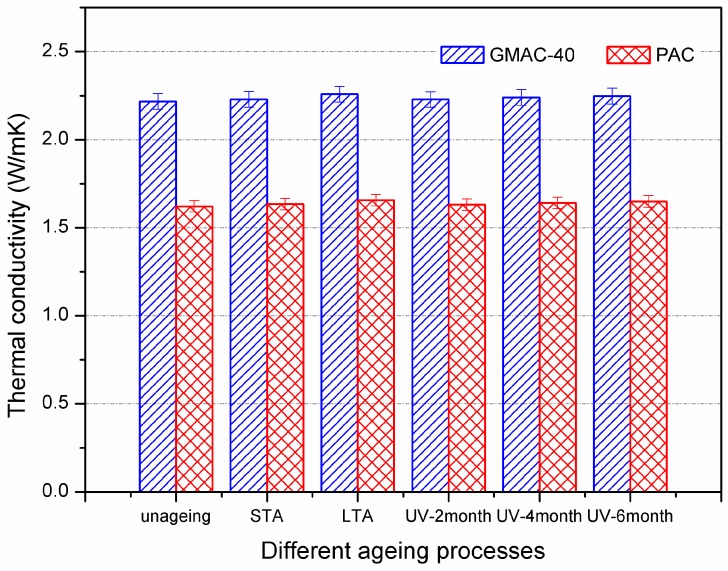
Effect of aging on thermal conductivity of control and conductive asphalt mixtures.

The asphalt can be easily aged under the heat, sunlight, oxygen, or combination of these factors, leading to changes in component, structure, and mechanical properties of asphalt. This might consequently induce some variation of thermal properties of asphalt concrete. Figure 10 illustrates the effect of aging on thermal conductivity of asphalt mixtures. The thermal conductivity, thermal diffusivity, and specific heat increased when prolonging the aging process. It is recognized that aging would increase the proportion of the colloid and asphaltene of asphalt, while decreasing the proportion of saturates and aromatics. Consequently, such variation of chemical components leads to changes in thermal properties of asphalt mixture. 

However, the differences in thermal conductivity, thermal diffusivity, and specific heat were less than 2% between aging specimens and control specimens, even for standard long-term aging or 6-month UV aging. This is because aging hardly affects the thermal properties of aggregate and filler, which account for more than 90% by weight of the asphalt mixture. The difference in thermal properties of asphalt could not result in a significant change in the corresponding performance of asphalt mixture, which is in accordance with the results in Table 4. This indicated that aging of asphalt is not a critical factor that affects the thermal properties of asphalt mixtures over a long term.

## 4. Conclusions

The effect of material composition and environmental conditions on thermal properties of conductive asphalt concrete for asphalt solar collector were investigated in this study. Based on the research results, the following conclusions can be drawn:

1. From the material composition of view, thermal properties of asphalt concrete are strongly dependent on the thermal properties of mineral aggregate. The effect of asphalt types is not significant due to its low proportion. Conductive filler and mineral aggregate with high thermal conductivity are recommended to prepare asphalt concrete for asphalt solar collector.
2. Environmental conditions, including temperature, water, freezing–thawing, and aging can affect the thermal characteristics of asphalt concrete. Among all the factors, the freezing–thawing effect is the most significant, due to volume expansion and bonding degradation of asphalt concrete.

Thermal properties of asphalt concrete are critical for accurately predicting the temperature distribution of pavement and evaluating the energy efficiency of asphalt solar collector. Therefore, the evolution of thermal properties of asphalt concrete is necessary to be considered at the very beginning before asphalt solar collector design. Further study is recommended to focus on the coupling effects of traffic loading and environmental factors on thermal characteristics of conductive asphalt concrete.

## Figures and Tables

**Table 1:** Pysical properties of different types of asphalt binders.

Indexes	Standard	AH-70	AH-90	SBS
Softening point (°C)	ASTM D36	47.2	46.3	53.6
Penetration (dmm)	ASTM D5	73	87	48.1
Viscosity (Pa·s)	ASTM D4402	0.59	0.32	1.28

**Table 2:** Physical properties of different types of aggregates.

Indexes	Standard	Basalt	Granite	Diabase	Limestone	Dacite	BOF Slag	Demolition Waste
Bulk S.G.	ASTM C127	2.842	2.691	2.953	2.716	2.709	3.197	2.591
Apparent S.G.	ASTM C127	2.887	2.723	2.970	2.754	2.740	3.396	2.623
Water absorption (%)	ASTM C127	0.4	0.6	0.3	0.8	0.4	2.5	6.9
Los Angeles abrasion (%)	ASTM C131	16.8	21.6	16.6	22.1	12.1	13.1	33.6
Volume expansion (%)	BS EN1744-1	N/A	N/A	N/A	N/A	N/A	1.3	N/A
Free lime content (%)	BS EN1744-1	N/A	N/A	N/A	N/A	N/A	2.3	N/A

BOF: basic oxygen furnace; S.G.: specific gravity.

**Table 3:** Thermal properties of different types of asphalt binders.

Asphalt	Thermal Conductivity (W/m·K)	Thermal Diffusivity (mm^2^/s)	Specific Heat (MJ/m^3^·K)
AH-70	0.169	0.123	1.374
AH-90	0.177	0.128	1.383
SBS	0.191	0.124	1.540

**Table 4:** Thermal properties of asphalt mixtures prepared by different asphalt binders.

Asphalt	Thermal Conductivity (W/m·K)	Thermal Diffusivity (mm^2^/s)	Specific Heat (MJ/m^3^·K)
AH-70	1.548	0.744	2.081
AH-90	1.597	0.759	2.105
SBS	1.622	0.748	2.166

**Table 5:** Thermal properties of different asphalt concrete.

Asphalt	Thermal Conductivity (W/m·K)	Standard Deviation (W/m·K)	Thermal Diffusivity (mm^2^/s)	Standard Deviation (mm^2^/s)	Specific Heat (MJ/m^3^·K)	Standard Deviation (MJ/m^3^·K)
Basalt	1.621	0.0465	0.774	0.0215	2.096	0.0719
Granite	1.781	0.0396	1.017	0.0334	1.751	0.0935
Diabase	1.717	0.0544	0.810	0.0362	2.120	0.0824
Limestone	1.594	0.0366	0.710	0.0342	2.246	0.0892
Dacite	2.123	0.0526	0.949	0.0298	2.238	0.0686
BOF slag	1.612	0.0567	0.812	0.0362	1.985	0.0757
Demolition	1.560	0.0588	0.871	0.0374	1.791	0.0682
